# The AREB transcription factor SaAREB6 promotes drought stress-induced santalol biosynthesis in sandalwood

**DOI:** 10.1093/hr/uhae347

**Published:** 2024-12-17

**Authors:** Sen Meng, Na Lian, Fangcuo Qin, Shuqi Yang, Dong Meng, Zhan Bian, Li Xiang, Junkun Lu

**Affiliations:** State Key Laboratory of Tree Genetics and Breeding, Research Institute of Tropical Forestry, Chinese Academy of Forestry, 682 Guangshan Road, Tianhe District, Guangzhou 510520, Guangdong, China; State Key Laboratory of Tree Genetics and Breeding, College of Biological Sciences and Technology, Beijing Forestry University, 35 Qinghuadong Road, Haidian District, Beijing 100083, China; State Key Laboratory of Tree Genetics and Breeding, Research Institute of Tropical Forestry, Chinese Academy of Forestry, 682 Guangshan Road, Tianhe District, Guangzhou 510520, Guangdong, China; State Key Laboratory of Tree Genetics and Breeding, Research Institute of Tropical Forestry, Chinese Academy of Forestry, 682 Guangshan Road, Tianhe District, Guangzhou 510520, Guangdong, China; College of Forestry, Beijing Forestry University, 35 Qinghuadong Road, Haidian District, Beijing 100083, China; Guangdong Provincial Key Laboratory of Applied Botany, South China Botanical Garden, Chinese Academy of Sciences, 723 Xingke Road, Tianhe District, Guangzhou 510650, China; State Key Laboratory of Tree Genetics and Breeding, Research Institute of Tropical Forestry, Chinese Academy of Forestry, 682 Guangshan Road, Tianhe District, Guangzhou 510520, Guangdong, China; State Key Laboratory of Tree Genetics and Breeding, Research Institute of Tropical Forestry, Chinese Academy of Forestry, 682 Guangshan Road, Tianhe District, Guangzhou 510520, Guangdong, China

## Abstract

Sandalwood (*Santalum album*), a culturally significant and economically valuable horticultural species, is renowned for its heartwood and essential oils enriched with sesquiterpene compounds such as santalol. Despite progress in elucidating the biosynthetic pathway of these valuable metabolites, the transcriptional regulation of this process, particularly under abiotic stress conditions, remains largely unexplored. Under drought conditions, we observed a marked increase in *SaAREB6* expression, paralleled by elevated levels of santalols. Moreover, we identified *SaCYP736A167*, a cytochrome P450 mono-oxygenase gene, as a direct target of SaAREB6. Using electrophoretic mobility shift assays (EMSAs), microscale thermophoresis assays (MSTs), and dual luciferase assays (DLAs), we validated the precise and specific interaction of SaAREB6 with the promoter region of *SaCYP736A167*. This interaction leads to the upregulation of *SaCYP736A167*, which in turn catalyzes the final steps in the conversion of sesquiterpene precursors to santalols, thereby reinforcing the connection between SaAREB6 activity and increased santalol production during drought. Collectively, our work illuminates the previously uncharacterized role of SaAREB6 in orchestrating a transcriptional regulation that facilitates drought-induced santalol biosynthesis in sandalwood, presenting opportunities for genetic engineering strategies to improve heartwood and essential oil yields in this economically vital species.

## Introduction

Indian Sandalwood (*Santalum album* L.), an evergreen horticultural species, is native to the tropical mountainous regions [1]. Widely acknowledged as the ‘royal tree’, *S. album* holds a deeply intertwined place within Buddhist culture and is globally esteemed as the second most valuable and expensive tree species, following closely behind African blackwood (*Dalbergia melanoxylon*) [2–4]. The heartwood and essential oils of *S. album* constitute its primary economic significance [5]. Specifically, the heartwood is extensively utilized in crafting high-end sculptures and furniture, while its powdered form, known as agarbatti, serves as a crucial component in incense preparation [3, 6]. Additionally, the essential oils derived from *S. album* are highly esteemed within the perfume industry due to their distinctive aromatic qualities and exceptional fixative properties, rendering them invaluable ingredients in the formulation of fragrances [1].

**Figure 1:**
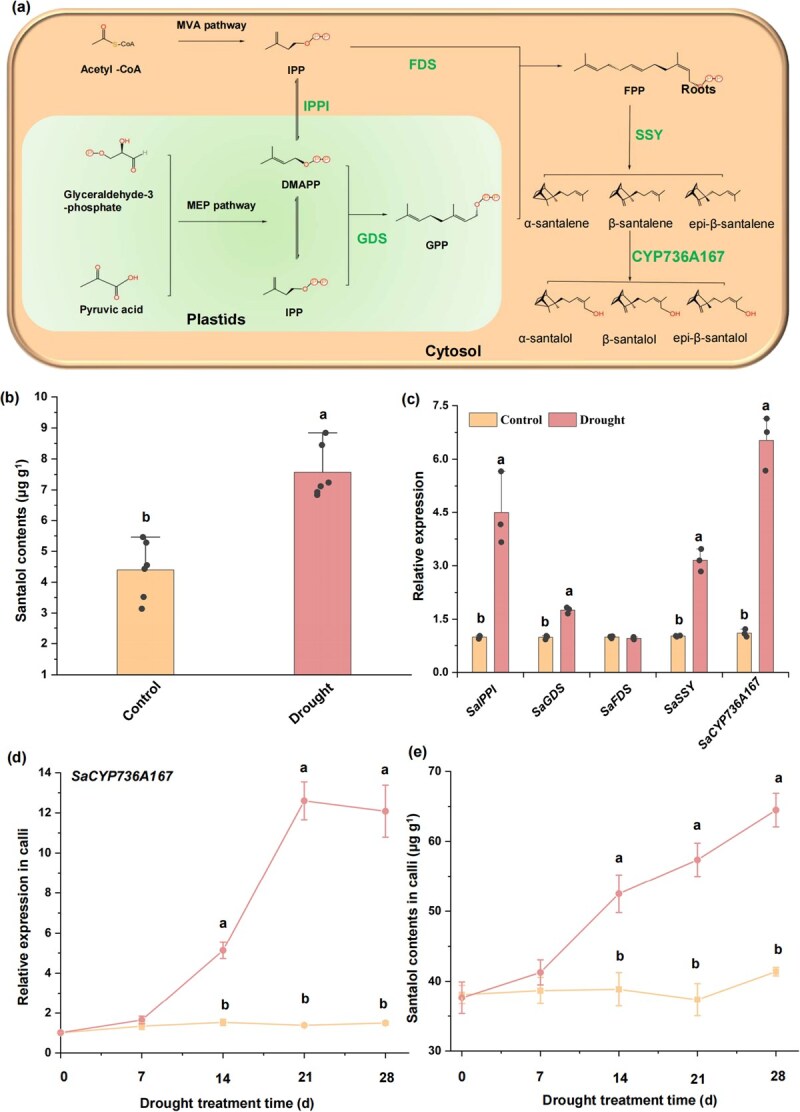
Drought stress regulates santalol biosynthesis in *S. album*. (a) The biosynthesis pathway of santalol in *S. album*. Abbreviations: MVA, mevalonate acid; MEP, methylerythritol 4-phosphate; DMAPP, dimethylallyl diphosphate; IPP, isopentenyl pyrophosphate; GPP, geranyl diphosphate; FPP, farnesyl diphosphate; IPPI, isopentenyl diphosphate isomerase; GDS, geranyl diphosphate synthase; FDS, farnesyl diphosphate synthase; SSY, santalene synthase. (b) Drought stress induces santalol biosynthesis in sandalwood seedlings. (c) Relative expression levels of key genes involved in the santalol biosynthesis pathway (*SaIPPI*, *SaGDS*, *SaFDS*, *SaSSY,* and *SaCYP736A167*) in response to drought stress. The results were based on three biological replicates and three technical replicates. (d) Relative expression levels of *SaCYP736A167* in response to simulated drought treatment in sandalwood calli. The results were based on three biological replicates and three technical replicates. (e) Santalol contents in response to simulated drought treatment in sandalwood calli. Means with different letters are significantly different (*P* < 0.05).

The primary constituents of sandalwood essential oil, comprising a notable array of sesquiterpene olefins and alcohols, are α-santalol and β-santalol [5]. Sandalwood oil holds a prominent position among the most extensively traded essential oils globally, and many studies have been devoted to clarifying the santalol biosynthetic pathway, which has been almost completely elucidated (Fig. 1a) [7–10]. The initial step in the biosynthetic pathway entails the head-to-tail condensation of isopentenyl pyrophosphate (IPP) to yield geranyl pyrophosphate (GPP), a process catalyzed by the enzyme geranyl diphosphate synthase (GDS) [11–13]. Subsequently, a second condensation reaction occurs, mediated by the enzyme farnesyl diphosphate synthase (FDS), resulting in the conversion of GPP into farnesyl pyrophosphate (FPP) [12]. Santalene synthase catalyzes FPP into a mixture of cyclic sesquiterpenes, such as α-santalene, β-santalene, and epi-β-santalene, and is further hydroxylated to α-santalol, β-santalol, and epi-β-santalol by cytochrome P450 mono-oxygenase (SaCYP736A167) [8, 10, 14].

Despite the clarification of the santalol biosynthetic pathway, the transcriptional regulation mechanisms governing this process remain largely unexplored. Notably, the synthesis and emission of terpene compounds are controlled by environmental stimuli, exhibiting both spatial and temporal regulation [15, 16]. For example, a series of studies have illuminated the regulatory role of diverse transcription factors (TFs) in terpene synthase (TPS) genes, including NAC [17], APETALA2/ethylene response factors (AP2/ERF) [18], ARF [19], MYB [20], and MYC [21], indicating their significance in modulating terpene production. Similarly, the synthetase genes involved in the methylerythritol phosphate pathway are also subject to transcriptional control by various factors [15, 22–24]. In contrast, the regulatory mechanisms underlying the mevalonate (MVA) pathway have received limited attention. One notable example is the influence of Glycoalkaloid Metabolism 9 (GAME9) on HMGR expression in tomato and potato, albeit without direct evidence of their interaction [25]. The TFs AREB/ABF/ABI5 are classified as basic leucine zipper proteins, and their functionalities encompass modulation of plant hormonal responses and stress adaptation [26, 27]. AREB/ABF/ABI5 TFs can bind to *cis*-acting abscisic acid (ABA)-responsive element (ABRE; PyACGTGGC) and activate the expression of ABA-dependent genes under various stress. For instance, ABF1 has been shown to be a type of gene that responds to cold and low temperature while ABF2/3/4 are primary TFs that cooperatively regulate ABRE-dependent gene expression for ABA signaling under conditions of water stress [28–30]. However, the specific pathway through which AREB factors modulate terpenoid production in sandalwood remains unexplored.

Drought stress conditions induce a wide range of impacts on growth and development, including perturbations in the production of secondary metabolites, particularly terpenes [31]. For instance, drought stress has been shown to profoundly elevate the emission of volatile terpenoids in Masson pine (*Pinus massoniana* L*.*) [32]. Additionally, in certain plant species such as rosemary (*Rosmarinus officinalis* L.), Holm oak (*Quercus Ilex* L.), and spearmint (*Mentha spicata* L.), drought stress acts as a stimulus, enhancing the synthesis of terpenes [33, 34]. Furthermore, research has demonstrated that under severe drought conditions, the total monoterpene content increases by 39% in Scots pine seedlings and 35% in Norway spruce seedlings, highlighting the complex interplay between drought stress and terpene production [35]. In *S. album*, drought stress triggers a considerable accumulation of osmotic adjustment substances, which is indicative of a substantial synthesis of secondary metabolites, potentially including terpenes [36].

Several field experiments have been undertaken in *Santalum* species to stimulate the biosynthesis of essential oils and the development of heartwood. Notably, stem injection with plant growth regulators (PGRs) has been demonstrated to effectively promote heartwood formation in *S. album* [37]. Additionally, H_2_O_2_ and benzyladenine have been reported to favorably influence the synthesis of sandal oil [38]. Despite the acknowledged significance of elucidating the responses of sandalwood to diverse environmental stimuli, such as drought, the molecular mechanisms underpinning the biosynthesis of santalol have remained largely uncharted territories.

To elucidate the molecular mechanisms underlying the biosynthesis of santalol in sandalwood, particularly in response to drought stress, we identify the TF SaAREB6 as a pivotal positive regulator of this process. Specifically, SaAREB6 directly interacts with the promoter region of *SaCYP736A167*, thereby inducing its expression. Furthermore, we observed that overexpression of the *SaAREB6* gene leads to an enhanced accumulation of santalol in *S. album* calli, whereas RNAi-mediated downregulation of *SaAREB6* expression results in a decrease in santalol content. The functional roles of *SaAREB6* in mediating drought-induced santalol accumulation are thoroughly examined and discussed. Collectively, our findings offer novel insights into the regulatory mechanisms governing santalol biosynthesis under drought stress conditions in sandalwood. Additionally, our research simultaneously offers reference and guidance for studies on the regulation of secondary metabolites in horticultural crops, particularly those related to flower and fruit color, aroma compounds, and other associated substances.

## Results

### The cytochrome mono-oxygenase *SaCYP736A167* is responsive to drought stress in sandalwood

To investigate the role of drought stress in modulating santalol biosynthesis, a comparative analysis of santalol content in *S. album* seedlings subjected to drought treatment and nondrought controls was conducted. The leaves of plants under drought treatment displayed stressed phenotypes, including wilting and sagging (Supplementary Fig. S1). Our findings also indicate that drought treatment led to a significant accumulation of santalol in the roots of the seedlings compared to the controls (Fig. 1b). Further analysis revealed that drought treatment induced the expression of multiple genes involved in the santalol synthesis pathway, including *SaIPPI*, *SaGDS*, *SaFDS*, *SaSSY*, and *SaCYP736A167* (Fig. 1c). Notably, *SaCYP736A167*, which encodes the key and final enzyme catalyzing the synthesis of santalol, exhibited the most pronounced upregulation in response to drought treatment (Fig. 1c). ABA is an indicative hormone that usually mediates drought-responsive genes and thus was used to test the response of *SaCYP736A167.* ABA treatment significantly upregulated the transcript of *SaCYP736A167* (Supplementary Fig. S2). Additionally, when drought conditions were simulated in sandalwood calli, we observed a corresponding increase in the expression of *SaCYP736A167* and the content of santalol, further corroborating the positive correlation between drought stress and santalol biosynthesis (Fig. 1d and e).

### Drought-induced santalol content is partly dependent on *SaCYP736A167*

**Figure 2:**
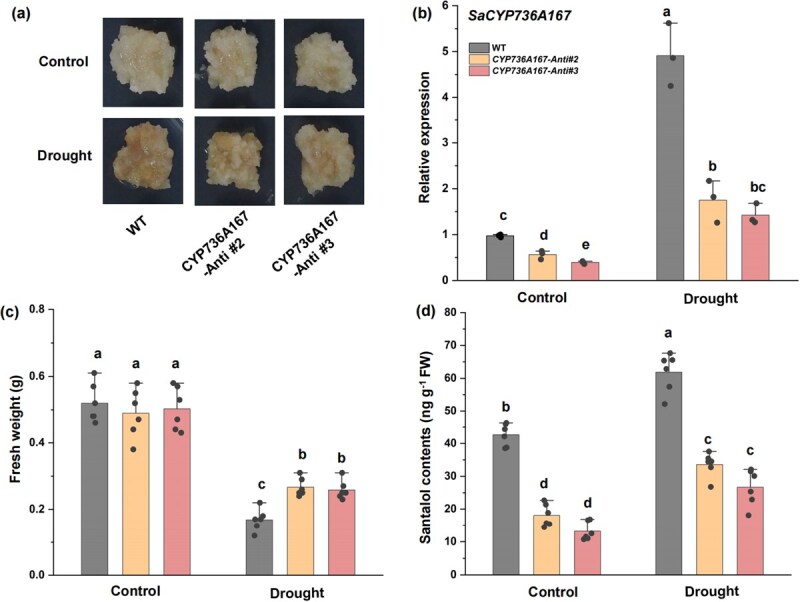
Drought-induced santalol biosynthesis is partly dependent on *SaCYP736A167*. (a) Drought stress phenotypes of *SaCYP736A167* RNAi sandalwood calli. WT and transgenic sandalwood calli (SaCYP736A167-Anti) were grown on media for 4 weeks. (b) Expression levels of *SaCYP736A167* in WT and transgenic sandalwood calli after drought treatment. (c) Fresh weight and (d) santalol contents of WT and transgenic sandalwood calli after drought treatment. Means with different letters are significantly different ( *P* < 0.05).

To substantiate the regulatory function of *SaCYP736A167* in drought-mediated santalol accumulation, we devised a simulated drought-triggered santalol induction system in sandalwood calli. Wild-type (WT) and *SaCYP736A167*-Antisense (*SaCYP736A167*-Anti) sandalwood calli were utilized for the purpose of subjecting them to simulated drought treatments (Fig. 2a and Supplementary Fig. S3). Upon induction of drought stress, the expression of *SaCYP736A167* was observed to be upregulated in the sandalwood calli (Fig. 2b). Under nondrought conditions, no significant differences in fresh weight were discernible between the WT and *SaCYP736A167*-Anti lines (Fig. 2c). However, under drought treatment, the fresh weight of the *SaCYP736A167*-Anti lines was markedly higher compared to the WT calli (Fig. 2c). Notably, the drought-induced biosynthesis of santalol was significantly diminished in the *SaCYP736A167*-Anti lines (Fig. 2d), underscoring the crucial role of *SaCYP736A167* in drought-stimulated santalol production.

### Expression and genetic analysis of SaAREB6

**Figure 3:**
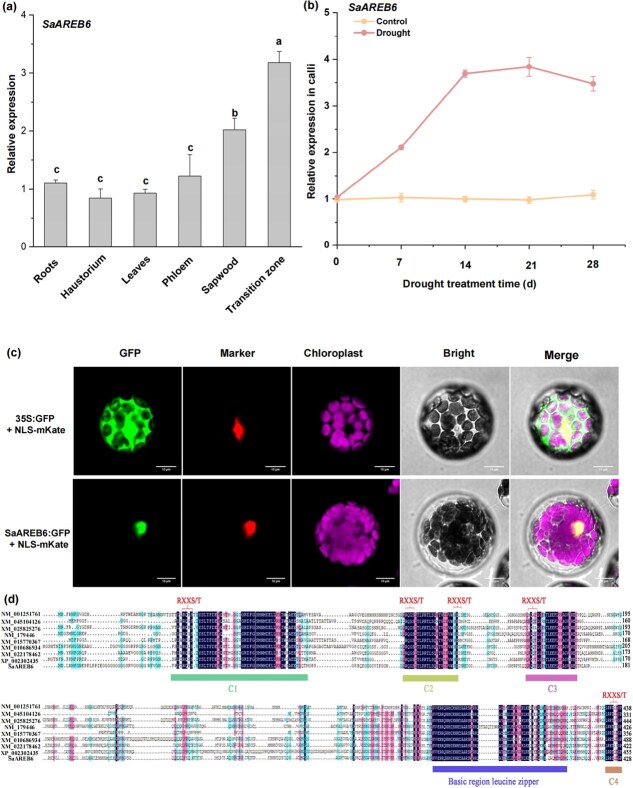
Identification analysis of *SaAREB6*. (a) Expression levels of *SaAREB6* in different tissues of sandalwood. (b) Relative expression levels of *SaAREB6* in response to simulated drought treatment in sandalwood calli. The results were based on three biological replicates and three technical replicates. (c) Subcellular localization of SaAREB6. (d) Multiple alignment of the amino acid sequences of SaAREB6 and other plant AREB proteins.

To uncover potential TFs capable of recognizing and interacting with the *SaCYP736A167* promoter, we cloned the promoter region spanning 2000 bp upstream of the *SaCYP736A167* gene and inserted it into the pAbAi vector. Based on the screening results, we have identified several candidate TFs, including AREB6, that potentially regulate the expression of the SaCYP736A167 gene (Supplementary Table S1). A further analysis of the *SaCYP736A167* promoter revealed various *cis*-regulatory elements, including two ABRE binding site sequences (CACGTG) related to AREB TFs (Supplementary Table S2). Thus, we hypothesized that SaAREB6 could specifically bind to these motifs within the *SaCYP736A167* promoter. We found that *SaAREB6* was highly enriched in the transition zone, aligning consistently with the pattern observed for *SaCYP736A167* (Fig. 3a and Supplementary Fig. S4). The expression of *SaAREB6* was also noticeably induced by drought treatment (Fig. 3b). Confocal laser scanning microscopy revealed that the control construct was expressed throughout the cell (Fig. 3c). In contrast, the fluorescence signal of the SaAREB6–GFP fusion protein overlapped with that of the cell nuclear localization marker (NLS-mKate) (Fig. 3c). Structural analysis of SaAREB6 revealed the presence of three conserved domains (designated C1, C2, and C3) at its N-terminal region, and a conserved domain (C4) at the C-terminal end (Fig. 3d). Notably, these four conserved domains harbor phosphorylation motifs (RXXS/T), indicative of potential post-translational modifications (Fig. 3d). Additionally, the C-terminal region encompasses a highly conserved basic leucine zipper (bZIP) structure which facilitates DNA binding and protein–protein interactions (Fig. 3d).

### SaAREB6 directly activates the transcription of *SaCYP736A167* to increase its expression

**Figure 4:**
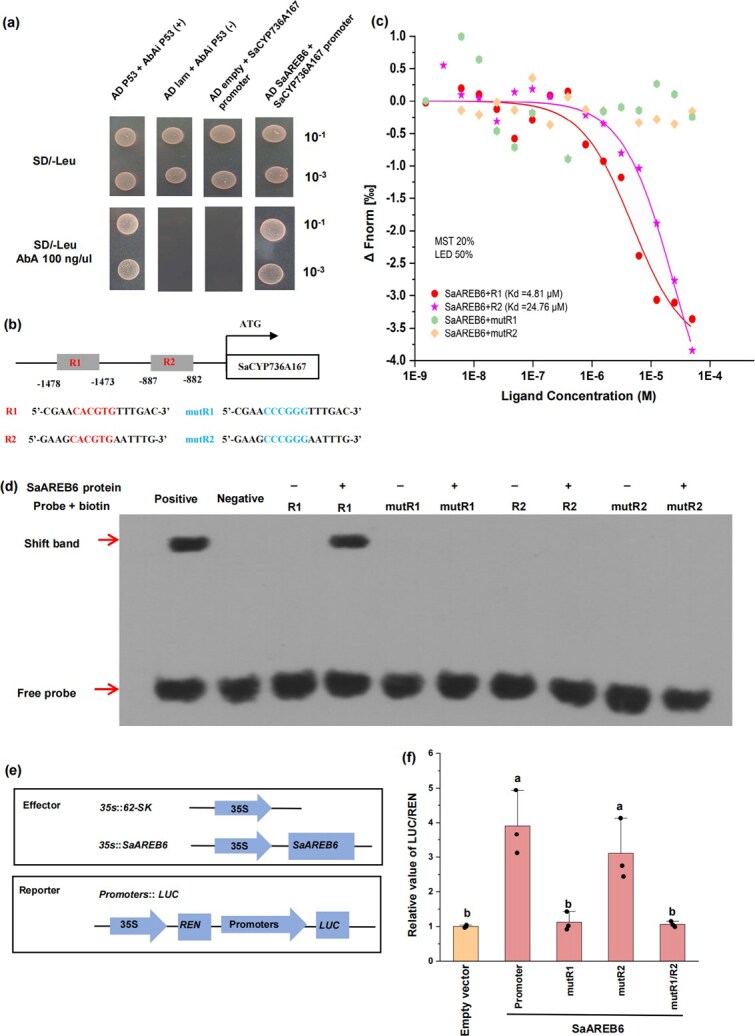
Regulatory effects of SaAREB6 on the *SaCYP736A167* gene. (a) Y1H assays showing that SaAREB6 interacted with the *SaCYP736A167* promoter in yeast. (b) Schematic diagram of the two ABRE binding site *cis* elements (R1 and R2) in the *SaCYP736A167* promoter. The elements in which the 5′-CACGTG-3′ motif was also replaced with the 5′-CCCGGG-3′ motif (mutR1 and mutR2). (c) MST assays showing that SaAREB6 binds to the *SaCYP736A167* promoter. (d) EMSA showing that SaAREB6 directly bound to the *SaCYP736A167* promoter at site I *in vitro*. (e) Schematic representation of the effector and reporter constructs used for the DLA. (f) Regulatory effects of SaAREB6 on the promoter of the *SaCYP736A167* gene, as determined using DLAs. Means with different letters are significantly different (*n* = 3, *P* < 0.05).

The yeast one-hybrid (Y1H) results revealed a strong interaction between SaAREB6 and promoters of *SaCYP736A167* (Fig. 4a)*.* We also constructed two mutant promoter regions of *SaCYP736A167* (mutR1 and mutR2), in which the CACGTG motifs were mutated to CCCGGG, and microscale thermophoresis (MST) analysis (Fig. 4b, c). Our results demonstrated that SaAREB6 bound to the R1 and R2 regions of the *SaCYP736A167* promoter with binding constants of 4.81 and 24.76 μM, respectively, whereas no binding was observed with the mutant promoters (Fig. 4c). The direct interaction between SaAREB6 and the *SaCYP736A167* promoter was further validated *in vitro* using electrophoretic mobility shift assays (EMSAs) (Fig. 4d). Specifically, SaAREB6 caused a pronounced upward shift in the band corresponding to the *SaCYP736A167* promoter fragment containing the CACGTG motifs, indicating robust DNA–protein binding (Fig. 4d). However, only the biotinylated probe R1, but not R2 or the mutated probes, was able to bind to the SaAREB6 (Fig. 4d). To assess the impact of SaAREB6 on the transcriptional activity of the *SaCYP736A167* promoter, transient transactivation assays were conducted in tobacco leaves. In these assays, the promoter regions of S*aCYP736A167,* along with artificially synthesized mutated variants (mutR1, mutR2, and mutR1/R2), were fused to luciferase (LUC) reporter genes (Fig. 4e). Our findings revealed that the promoter activity of *SaCYP736A167* was significantly enhanced (3.91-fold) upon transient transactivation of *SaAREB6* in tobacco leaves, compared to the control (Fig. 4f). Similarly, SaAREB6 also displayed a 3.11-fold activation effect on mutR2. However, mutR1 and mutR1/R2 lost their responsiveness to SaAREB6-mediated activation (Fig. 4f). These results collectively support the specific binding of SaAREB6 to the R1 region of the *SaCYP736A167* promoter and its role in enhancing the expression of *SaCYP736A167 in vitro*.

### SaAREB6 promotes santalol biosynthesis via *SaCYP736A167*

**Figure 5:**
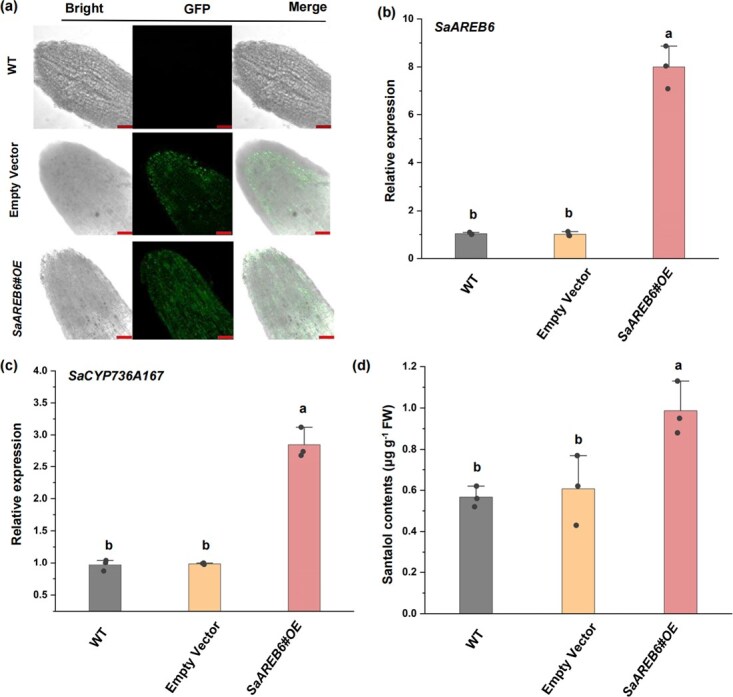
Transient expression assays showing that SaAREB6 promotes *SaCYP736A167* expression and santalol biosynthesis in sandalwood. (a) GFP fluorescence in roots infiltrated with *SaAREB6* driven by the CaMV 35S promoter. (b, c) Relative expression of *SaAREB6* and *SaCYP736A167* in roots of plants. (d) Santalol content in roots infiltrated with vectors. The roots of 30 plants were combined to form a mixed sample. Means with different letters are significantly different (*n* = 3, *P* < 0.05).

Sandalwood, as a perennial woody plant species, poses challenges in achieving stable genetic transformation due to its inherent recalcitrance. To overcome this limitation and facilitate preliminary functional assessment of SaAREB6 *in vivo*, a rapid transient overexpression assay was adopted (Fig. 5a). The transient expression assays involved vacuum-infiltrating 8-month-old *S. album* plantlets, transferring them to Hoagland’s nutrient solution, incubating them overnight in the dark, and finally placing them in a growth room. *Agrobacterium tumefaciens* GV3101 cells containing the empty vector (pBWA(V)HS, *35S*::*GFP*) and *35S*::*SaAREB6*::*GFP* vector were separately infiltrated into the roots. Following a 3-day postinfiltration period, the santalol content was quantitatively analyzed (Fig. 5a). The results revealed a notable upregulation in the endogenous mRNA expression of *SaCYP736A167* upon the introduction of *SaAREB6* (Fig. 5b). Furthermore, the santalol content in roots infiltrated with *SaAREB6* was found to be significantly elevated, reaching a level of 0.99 μg g^−1^, in comparison to the roots infiltrated with the empty vector, which had a santalol content of 0.61 μg g^−1^ (Fig. 5c and d). These findings provide compelling evidence that the transient overexpression of *SaAREB6* effectively accelerates the synthesis of santalol in sandalwood.

**Figure 6:**
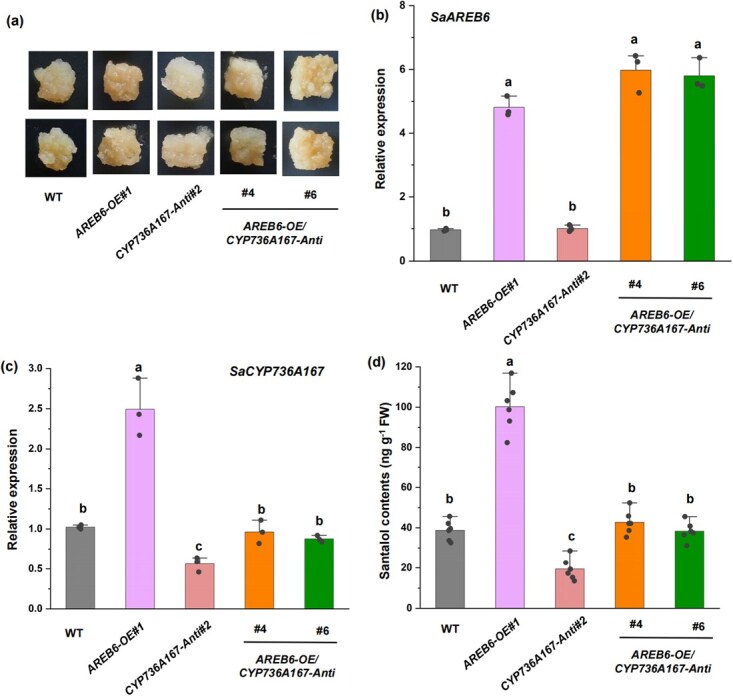
SaAREB6 promotes santalol biosynthesis via *SaCYP736A167* in transgenic sandalwood calli. (a) Phenotypes of 4-week-old WT and transgenic sandalwood calli (*SaAREB6*-OE, *SaAREB6* overexpression in sandalwood calli; *SaCYP736A167*-Anti, antisense suppression of *SaCYP736A167* in sandalwood calli; *SaAREB6-OE*/*SaCYP736A16*-Anti, overexpression of *SaAREB6* in the background of *SaCYP736A167* RNAi sandalwood calli). (b, c) Relative expression of *SaAREB6* and *SaCYP736A167* in WT and transgenic sandalwood calli. **(**d) Santalol content in WT and transgenic sandalwood calli. Means with different letters are significantly different (*P* < 0.05).

To further explore the mechanisms underlying the influence of SaAREB6 on drought-elicited santalol accumulation, we conducted a study in which *SaAREB6* was overexpressed (*SaAREB6*-OE) and subsequently employed to transform *S. album* calli, alongside the suppression of *SaCYP736A167* expression (*SaCYP736A167*-Anti) (Fig. 6a, b, and c). Our findings reveal that *S. album* lines overexpressing *SaAREB6* exhibited notably elevated levels of santalol compared to WT *S. album* calli (Fig. 6d). Importantly, when *SaCYP736A167* expression was repressed in the context of *SaAREB6*-OE *S. album* calli, there was a significant reduction in the *SaAREB6*-mediated enhancement of santalol accumulation (Fig. 6c and d). These observations suggest that the capacity of *SaAREB6* to promote santalol accumulation is likely to be partially dependent on *SaCYP736A167*.

### SaAREB6 plays an essential role in drought-induced santalol accumulation

**Figure 7:**
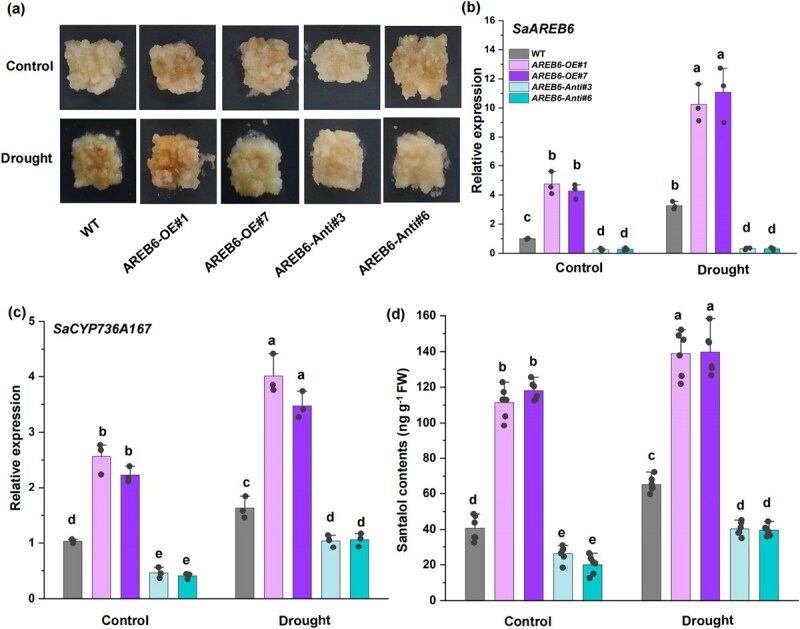
SaAREB6 plays an essential role in drought-induced santalol accumulation. (a) Phenotypes of WT and transgenic sandalwood calli grown on media supplemented with drought treatment for 4 weeks. (b, c) Expression levels of *SaAREB6* and *SaCYP736A167* in WT and transgenic sandalwood calli after drought treatment. (d) Santalol content of WT and transgenic sandalwood calli grown on media supplemented with drought treatment for 4 weeks. Means with different letters are significantly different ( *P* < 0.05).

Given that SaAREB6 appears to activate the transcription of *SaCYP736A167* and that *SaCYP736A167* plays an essential role in drought-induced santalol biosynthesis, we next asked whether *SaAREB6* affects santalol biosynthesis in response to drought stress in *S. album*. To gain further insights into the functional implications of *SaAREB6* in this process, we constructed overexpression (*SaAREB6*-OE) and antisense suppression (*SaAREB6*-Anti) vectors of *SaAREB6* and introduced them into *S. album* calli (Fig. 7a). Quantitative real-time polymerase chain reaction (RT-qPCR) analysis subsequently demonstrated that the expression of *SaCYP736A167* was upregulated in *SaAREB6*-OE lines compared to WT controls, while it was downregulated in *SaAREB6*-Anti lines (Fig. 7b and c). Notably, the overexpression of *SaAREB6* led to an enhancement of drought-induced santalol accumulation, whereas the antisense suppression of *SaAREB6* in *S. album* lines resulted in the opposite phenotype (Fig. 7d).

## Discussion

Drought has always been an important environmental factor and significantly impacts plant growth and productivity [39, 40]. This stressor elicits a cascade of physiological responses in plants, including decreased photosynthesis, altered gene expression patterns, and elevated concentrations of secondary metabolites [41, 42]. However, it is imperative to note that the observed augmentation in secondary metabolite levels in drought-stressed plants does not invariably signify an enhancement in their biosynthetic rates. Rather, this increase can be attributed to the reduced biomass accumulation, which, under equal biosynthetic rates, leads to an apparent elevation in metabolite concentrations per unit of biomass [43, 44]. Nonetheless, drought stress can also directly stimulate biosynthetic processes of secondary metabolites [45]. Here, we observed a pronounced induction of the expression of key genes involved in santalol biosynthesis in response to drought stress, including *SaCYP736A167*, in both *S. album* seedlings and sandalwood calli (Fig. 1). Notably, the drought-treated seedlings and calli exhibited significantly higher santalol accumulation compared to their nonstressed controls (Fig. 1b and e). Moreover, the suppression of *SaCYP736A167* expression in calli attenuated drought-induced santalol production, highlighting the pivotal role of this gene in mediating drought-responsive santalol biosynthesis (Fig. 2). Collectively, these results underscore the role of drought stress in promoting santalol production and emphasize the significance of transcriptional upregulation of genes in the santalol biosynthetic pathway.

In the context of drought stress in plants, the hormone ABA orchestrates intricate physiological and biochemical responses [46]. Upon the onset of drought conditions, ABA undergoes a rapid accumulation within plant tissues, where it interacts with the ABA receptor PYR1/PYL, eliciting conformational alterations that enable receptor recognition and binding of protein phosphatase 2C (PP2C) [47]. This process facilitates the release of the protein kinase SnRK2, which can then be activated through autophosphorylation or by additional protein kinases [48]. The activated SnRK2 subsequently phosphorylates conserved RXXS/T motifs, activating downstream AREB/ABF-type TFs [46, 49, 50]. These TFs specifically recognize and bind ABA-responsive elements within the promoter of target genes, thereby modulating their expression. Here, SaAREB6 emerges as a candidate TF implicated in the regulation of *SaCYP736A167* expression (Fig. 3). Direct evidence of SaAREB6’s regulatory role was provided by its physical interaction with CACGTG motifs in the *SaCYP736A167* promoter (Fig. 4). Notably, SaAREB6 exhibited robust expression levels in both the sapwood and the transition zone and was a typical drought-induced AREB TF (Fig. 3a). This observation suggests a potential link between *SaAREB6* and the biosynthesis of santalols in heartwood. These findings are in alignment with previous study, which demonstrated through RNA-seq data that the transcript abundance of the *SaAREB6* genes exhibited significantly higher expression levels in the transition region and underscore that SaAREB6 may play a crucial role in facilitating santalol accumulation [51].

Terpenoids encompass the most diverse and expansive category of plant secondary metabolites [52]. The biosynthetic pathways leading to terpenoid production are intricate and subject to modulation by various environmental stimuli, with drought stress being a prominent inducer in species such as *Bauhinia ungulata*, *Cupressus arizonica*, *Salvia officinalis*, and *Salvia dolomitica* [41, 43, 45, 53]. In the present study, we observed a correlation between drought-induced upregulation of the TF *SaAREB6* and enhanced expression of the *SaCYP736A167* gene (Fig. 5b and c). This finding was further validated through cotransformation phenotype detection analyses, indicating that *SaAREB6*-mediated santalol accumulation is partially contingent upon the activity of *SaCYP736A167* (Fig. 6). To elucidate the precise role of SaAREB6 in regulating santalol synthesis, we conducted overexpression and silencing experiments in *S. album* calli. Our results revealed that elevated expression of *SaAREB6* under drought stress conditions led to a marked increase in santalol accumulation, whereas antisense-mediated repression of *SaAREB6* resulted in a corresponding decrease in santalol levels (Fig. 7). These findings underscore the crucial regulatory function of *SaAREB6* in promoting santalol biosynthesis and highlight its potential as a target for genetic engineering strategies aimed at enhancing the efficient production of this valuable secondary metabolite.

## Conclusion

**Figure 8:**
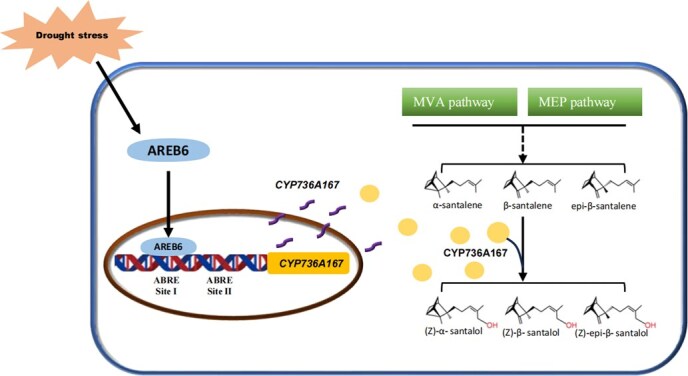
A possible model for the role of SaAREB6 in drought stress and santalol accumulation. SaAREB6 binds to the ABRE-motif siteIin, the promoter of *SaCYP736A167*, enhances the production of cytochrome P450 mono-oxygenase SaCYP736A167, and finally increases santalol accumulation.

In summary, our study has unveiled a novel AREB TF, SaAREB6, which contributes to drought-induced santalol biosynthesis in *S. album* (Fig. 8). Y1H assays, MSTs, dual luciferase assays (DLAs), and EMSAs suggested that SaAREB6 can recognize and regulate the promoter of *SaCYP736A167*, indicating that SaAREB6 has the ability to directly regulate santalol biosynthesis. We propose that, in the cultivation practices of sandalwood plantations, drought, as an environmental stimulus, can increase essential oil production by inducing transcripts of *SaAREB6* that act on the cytochrome mono-oxygenase SaCYP736A167.

## Materials and methods

### Plant materials and growth conditions

Sandalwood seedlings were cultivated in a greenhouse environment under controlled conditions of natural light, a temperature range of 20%–25°C, and a relative humidity of 75%. To ensure uniformity and mitigate potential edge effects, the seedlings were randomly rotated every 2 days and nourished with a modified Hoagland’s nutrient solution [54]. Subsequently, seedlings of uniform height (25 cm) were subjected to a drought stress treatment involving the withholding of water for a period of 4 weeks. Additionally, different tissues, including the haustorium, roots, leaves, phloem, sapwood, and transition zone, were harvested from 17-year-old sandalwood trees and immediately frozen in liquid nitrogen for the purpose of examining tissue-specific expression patterns (Supplementary Fig. S5).

Explants were harvested from seedlings located within a greenhouse environment and subsequently sanitized through the application of cotton swabs soaked in 75% ethanol. Following this initial cleaning step, the explants were immersed in a 0.1% (w/v) HgCl_2_ for 10 min. Subsequently, the explants underwent three to five consecutive rinses using sterile distilled water. Shoot segments, ~1.0 cm in length and containing a single node, were vertically inoculated onto a callus induction medium, specifically Murashige and Skoog (MS) medium supplemented with 1.0 mg/l of 2,4-dichlorophenoxyacetic acid (2,4-D). The resultant callus cultures of sandalwood were then subcultured onto the identical medium. All cultures were incubated in a growth room (16-h light/8-h dark, day/night temperature: 25°C/22°C). To investigate the effect of simulated drought on santalol accumulation, these callus cultures were subjected to treatment with 2% polyethylene glycol 6000 (PEG6000).

### Gene isolation, sequence analysis, and subcellular localization

The total RNA was extracted and purified employing the Omega reagent (R6827, Omega Bio-Tek, Norcross, GA, USA). The RNA was then evaluated by measuring the OD260/OD280 ratio using a NanoDrop 2000 Spectrophotometer (Thermo, West Palm Beach, FL, USA), while its integrity was assessed through electrophoresis. Subsequently, cDNA was synthesized from DNase-treated RNA using the PrimeScript RT reagent kit (DRR037S, Takara, Dalian, China). The specific cDNA sequence of *SaAREB6* was amplified via PCR. For comparative analysis, amino acid sequences were aligned using ClustalW (http://clustalw.ddbj.nig.ac.jp/), and a phylogenetic tree was constructed utilizing the maximum likelihood method implemented in MEGA 7.0 software (http://www.megasoftware.net/) [55]. Sequences from various plant species, including *Glycine max* (accession no. NM_001251761), *Hordeum vulgare* (accession no. XM_045104126), *Arachis hypogaea* (accession no. XM_025825276), *Arabidopsis thaliana* (accession no. NM_179446), *Oryza sativa* (accession no. XM_015770367), *Beta vulgaris* (accession no. XM_010686934), *Helianthus annuus* (accession no. XM_022178462), and *Populus trichocarpa* (accession no. XP_002302435), were retrieved from the National Center for Biotechnology Information (NCBI) database for inclusion in the analysis.

The full-length cDNA of *SaAREB6* was subcloned into the pBWA(V)HS vector (35S:*SaAREB6*-GFP). This construct was then transformed into Arabidopsis leaf protoplasts, following established protocols [56]. As a nuclear localization marker, NLS-mKate was employed, and the transformed protoplasts were subsequently visualized (Leica TCS SP8 instrument, Solms, Germany).

### Vector construction and genetic transformation

For the construction of *SaAREB6* overexpression (*SaAREB6*-OE), SaAREB6 knockdown (*SaAREB6*-Anti), and *SaCYP736A167* knockdown (*SaCYP736A167*-Anti) constructs, the respective coding sequences and interference fragments of *SaAREB6* and *SaCYP736A167* were inserted into a pBWA(V)HS vector. For the purpose of transforming sandalwood calli, 4-week-old WT sandalwood calli were cocultured with *A. tumefaciens* LBA4404 strains containing the respective constructs for a duration of 20 min. Following this, the calli were placed on MS media (adjusted to pH 5.8) supplemented with 1.0 mg/l of 2,4-D. Subsequently, the calli were transferred to selection media containing hygromycin (40 mg·l^−1^) to identify and select for successfully transformed sandalwood calli.

### Y1H

The promoter fragments of *SaCYP736A167* were cloned and inserted into the pAbAi vector, resulting in the AbAi-proSaCYP736A167 construct. Additionally, the full-length cDNA of *SaAREB6* was inserted into the pGADT7 vector, generating the AD-SaAREB6 construct. Subsequently, the AbAi-proSaCYP736A167 vector was cotransformed along with the AD-SaAREB6 construct. The resulting transformants were grown on SD/−Leu/AbA plates for a period of 3 days at 28°C.

### Protein expression and purification

*SaAREB6* was inserted into the PET-22b vector (NEB, Baverly, MA, USA) to enable their expression as His-tagged fusion proteins (designated as SaAREB6-His). These fusion proteins were expressed in Rosetta cells (DE3 strain, TransGen Biotech, Beijing, China). Following expression, the SaAREB6-His proteins were purified using amylose affinity chromatography.

### Microscale thermophoresis assay

The SaAREB6 protein was labeled with the red fluorescent dye NHS, utilizing the Monolith NT™ Protein Labeling Kit RED-NHS (2nd Generation; NanoTemper Technologies GmbH; Munich, Germany), adhering strictly to the manufacturer’s instructions. During the experiment, the concentration of SaAREB6 was maintained at a constant level of 20 nM, and the concentrations of the *SaCYP736A167* promoters were subjected to gradient dilution. Following a brief incubation period in MST buffer (150 mM NaCl, 20 mM Tris–HCl at pH 8.0), the samples were carefully loaded into MST-standard glass capillaries. The affinity measurements were conducted at a temperature of 25°C (50% LED power, 20% MST power). Each affinity measurement was replicated three times.

### Electrophoretic mobility shift assay

The promoter region of *SaCYP736A167* gene, which encompass the CACGTG motifs, were biotinylated by Biomed Company (Beijing, China). The probe sequences are listed in Supplementary Table S3. The cAMP receptor protein, when paired with the biotin-labeled lac promoter sequence (214 bp), served as the positive control, whereas its pairing with the unlabeled *lac* promoter DNA fragment constituted the negative control [57]. The EMSA experiments were conducted in triplicate using Lightshift Chemiluminescent EMSA Kit (Thermo Fisher Scientific, Waltham, MA, USA), with consistent results observed across all repetitions.

### Dual luciferase assays

The ORF of SaAREB6 was introduced into the pGreen II 0029 62-SK vector (SK) and the *SaCYP736A167* promoter fragments and artificially synthesized mutated variants were inserted into the pGreen II 0800-LUC vector using the EasyGeno Assembly Cloning Kit (Tiangen, Beijing, China). The primers were detailed in Supplementary Table S3. These recombinant vectors were then transformed into tobacco leaves utilizing the *A. tumefaciens* GV3101-mediated genetic transformation. For the quantification of LUC/REN activity, a luciferase detection kit (Promega, Madison, WI, USA) was employed. The experiments were conducted in triplicate.

### Transient expression assays using *S. album* plantlets

To preliminarily investigate the potential role of the *SaAREB6* gene in regulating santalol biosynthesis in sandalwood, an efficient transient overexpression system was employed to assess the *in vivo* function of SaAREB6 [40]. The full-length *SaAREB6* cDNA was cloned into the pBWA(V)HS vector under the control of the *35S* promoter. The resulting *35S*::*SaAREB6*::*GFP* was then transformed into *A. tumefaciens* strain GV3101. Prior to infiltration, the cultures were resuspended in a buffer solution (10 mM MES, 10 mM MgCl_2_, 5% sucrose, 0.05% Silwet L-77, and 200 mM acetosyringone (pH 5.8)), and were incubated at room temperature for 4–5 h. The transient expression assays were performed on 8-month-old *S. album* plantlets of similar size, using a vacuum infiltration method in a 10-l glass container. Following infiltration, the plantlets were transferred to Hoagland’s nutrient solution, incubated overnight in the dark, and then moved to a growth room for a period of 3 days. Subsequently, the roots from 30 plants within each group were pooled to create a mixed sample for further analysis of santalol content and endogenous *SaCYP736A167* expression levels.

### Determination of the santalol content

The quantification of santalol in sandalwood calli and roots was conducted following a previously established protocol [58]. In summary, 0.1 g of ground and dried calli or roots were subjected to metabolite extraction using 1 ml of hexane (Fisher Scientific, Waltham, MA, USA) via end-over-end shaking for 3 days. Following centrifugation at 2000 rpm for 5 min, the supernatant was subjected to evaporation to dryness. The dried residue was subsequently redissolved in 50 μl of hexane. Dodecane was added as an internal standard for gas chromatography–mass spectrometry (GC–MS) analysis. The GC–MS analysis was performed on a GC-2010 gas chromatograph (Shimadzu, Suzhou, China). The temperature parameters were as follows: the injector temperature was maintained at 250°C for 2 min, the ion source and MS interface temperatures were set at 250°C, and the oven temperature was programmed to increase from 50°C to 250°C at a rate of 5°C/min, held at 250°C for 2 min, further increased to 300°C at a rate of 5°C/min, and then held at 300°C for 20 min.

### Quantitative real-time PCR analysis

cDNA was subjected to dilution, and the subsequent reactions were carried out utilizing SYBR Green RT-qPCR Master Mix (Qiagen, Düsseldorf, Germany) in a total reaction volume of 20 μl. In parallel, the sandalwood *Actin* gene was detected and employed as an internal control. The primers were detailed in Supplementary Table S3.

### Statistical analysis

The data were analyzed using SPSS 26.0 software, with one-way ANOVA and Duncan’s multiple range test employed for statistical significance analysis.

## Supplementary Material

Web_Material_uhae347

## Data Availability

The supplementary material contains comprehensive data that substantiates the conclusions drawn from this study.
