# Supplementary figures and images for: The AREB transcription factor SaAREB6 promotes drought stress-induced santalol biosynthesis in sandalwood
Source: Hortic Res. 2024 Dec 17;12(3):uhae347. doi: 10.1093/hr/uhae347 (PMC11890025; doi:10.1093/hr/uhae347)

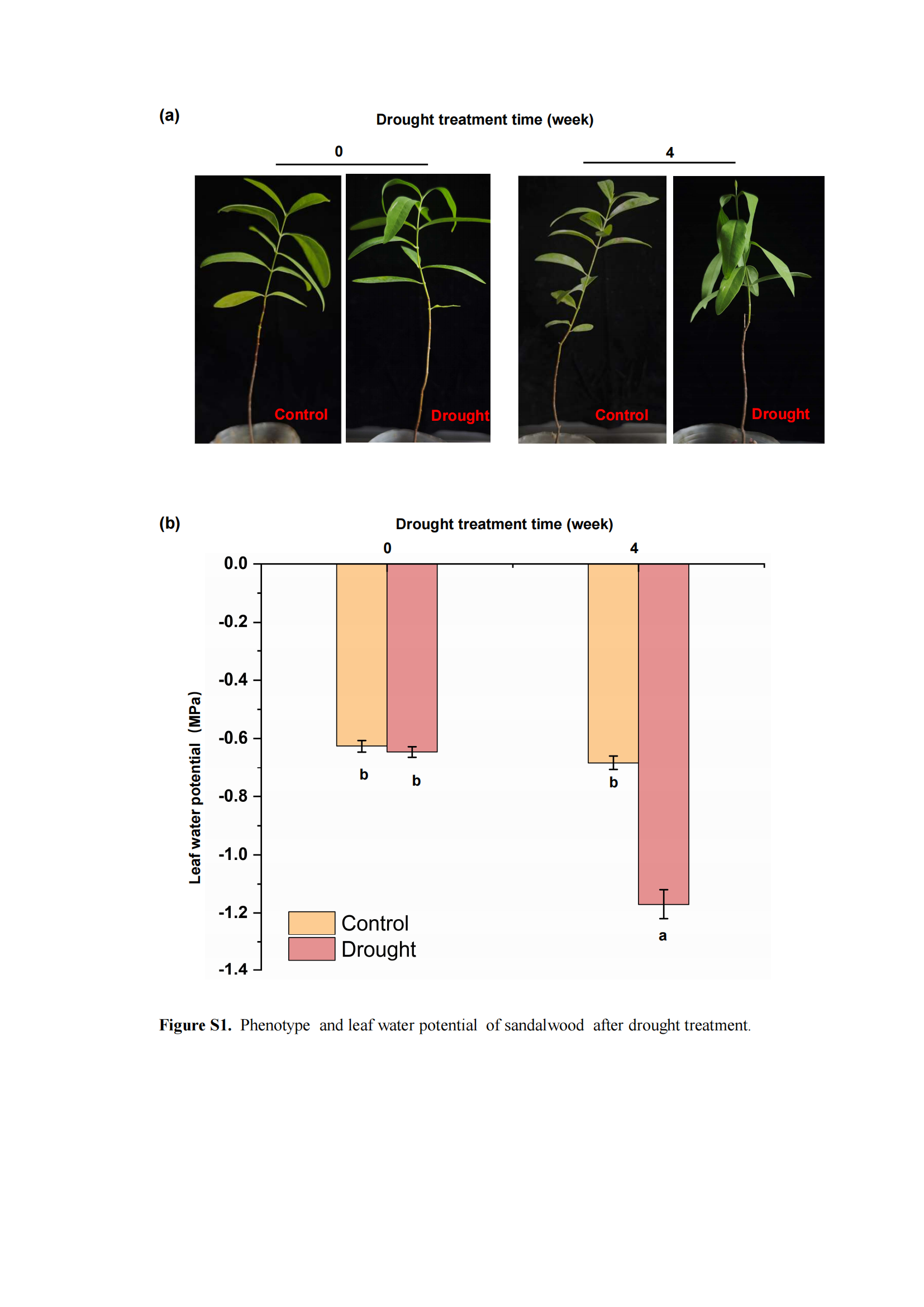

Supplement: Web_Material_uhae347 [file web_material_uhae347.zip › Fig S1.tif]

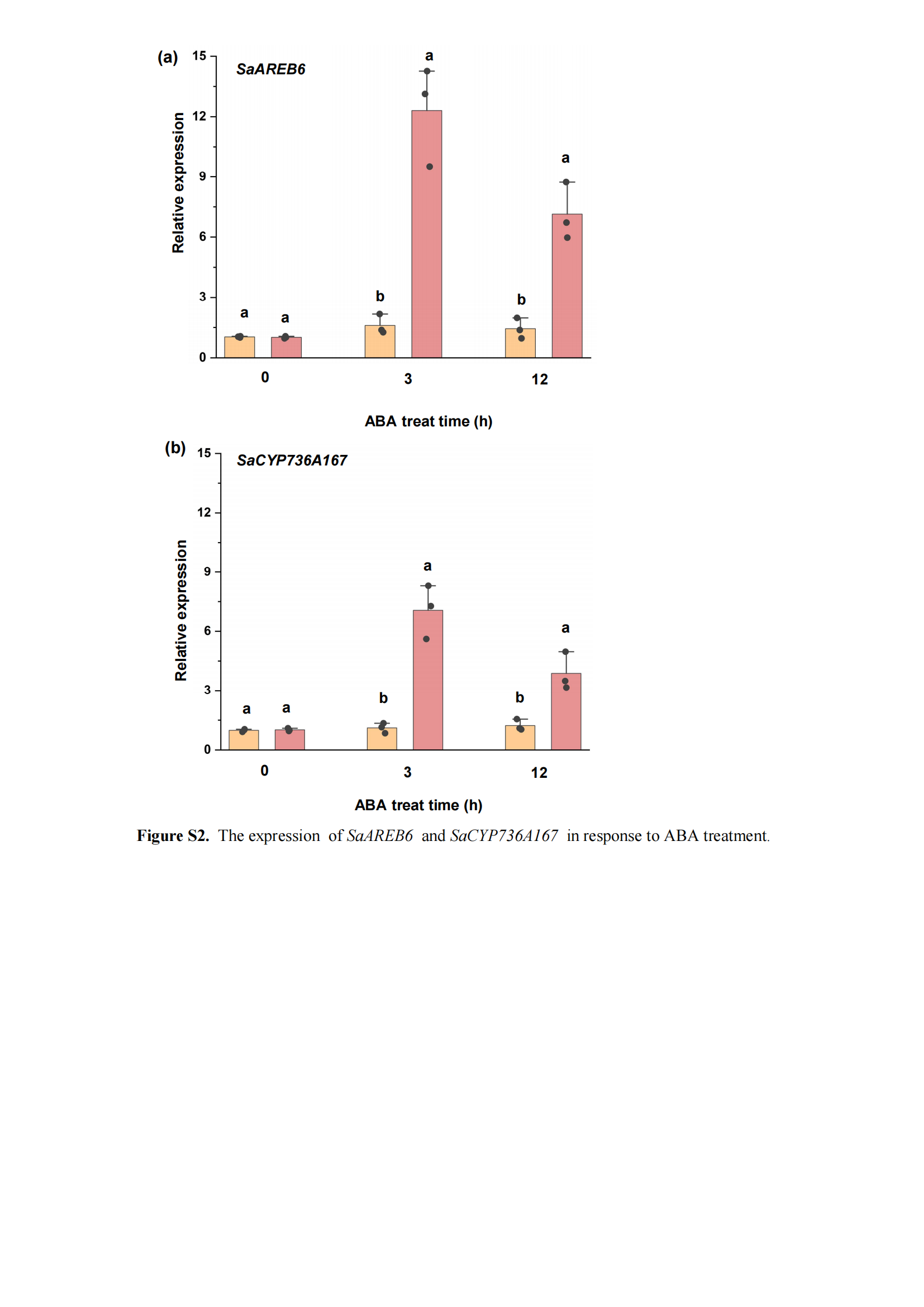

Supplement: Web_Material_uhae347 [file web_material_uhae347.zip › Fig S2.tif]

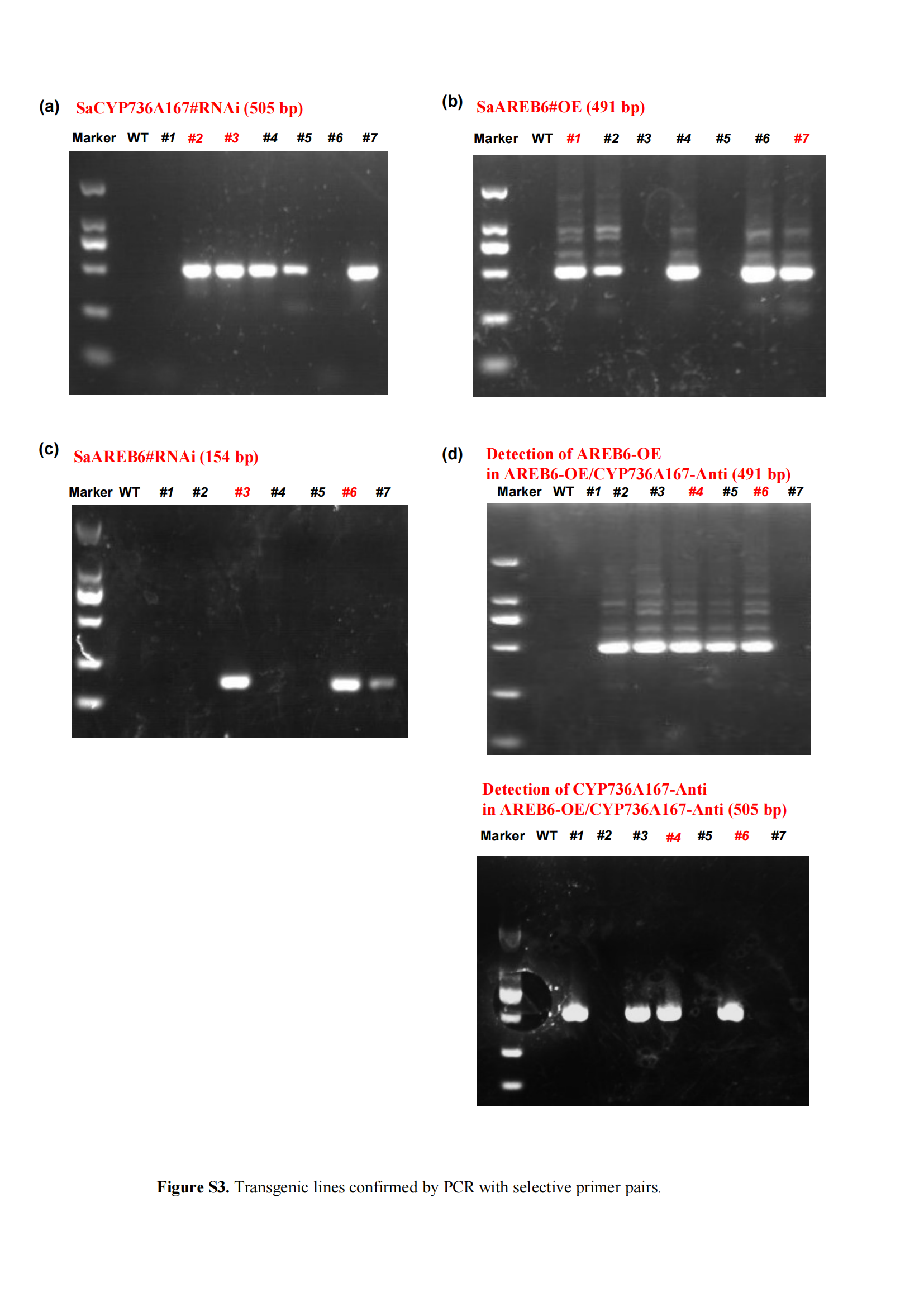

Supplement: Web_Material_uhae347 [file web_material_uhae347.zip › Fig S3.tif]

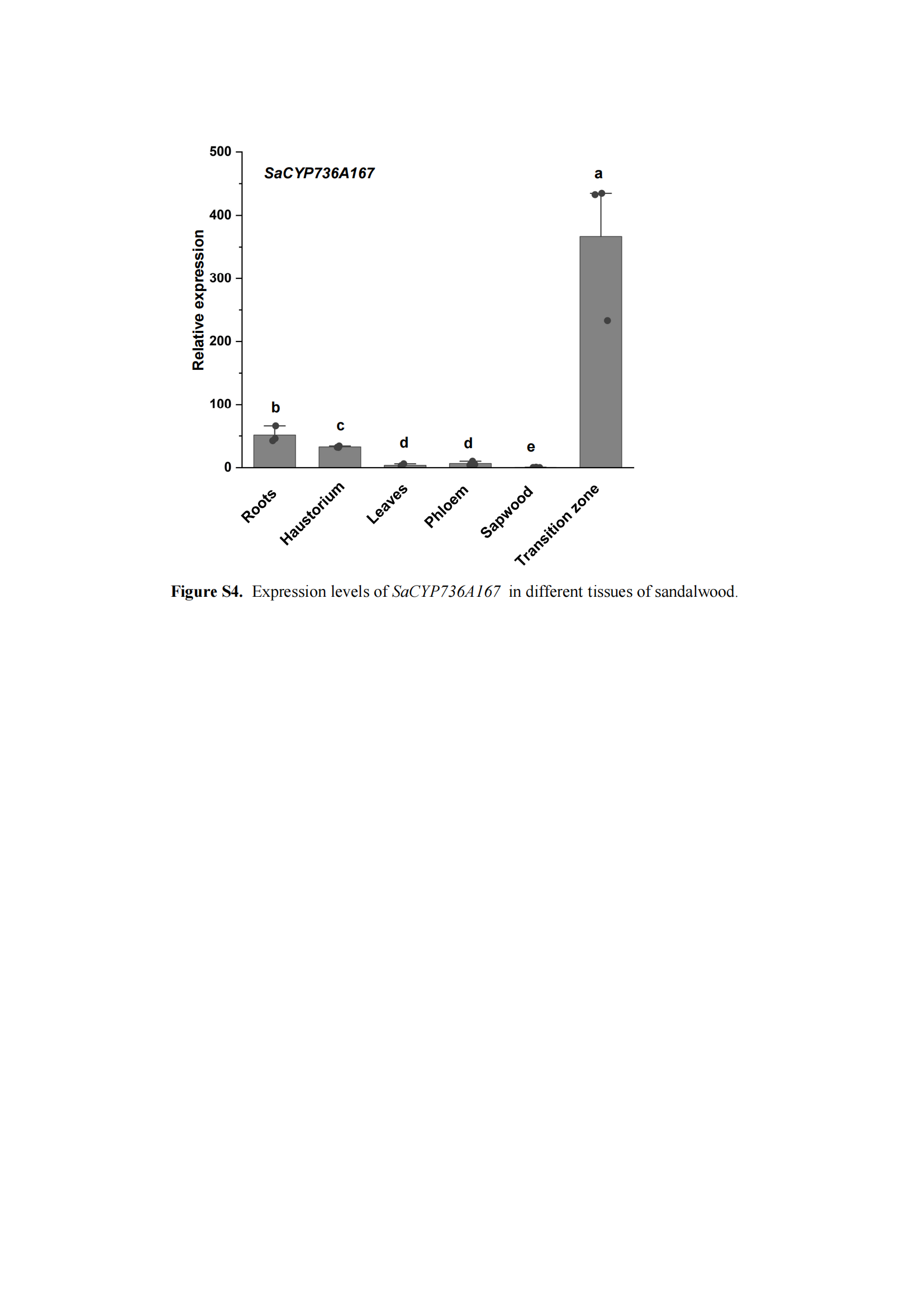

Supplement: Web_Material_uhae347 [file web_material_uhae347.zip › Fig S4.tif]

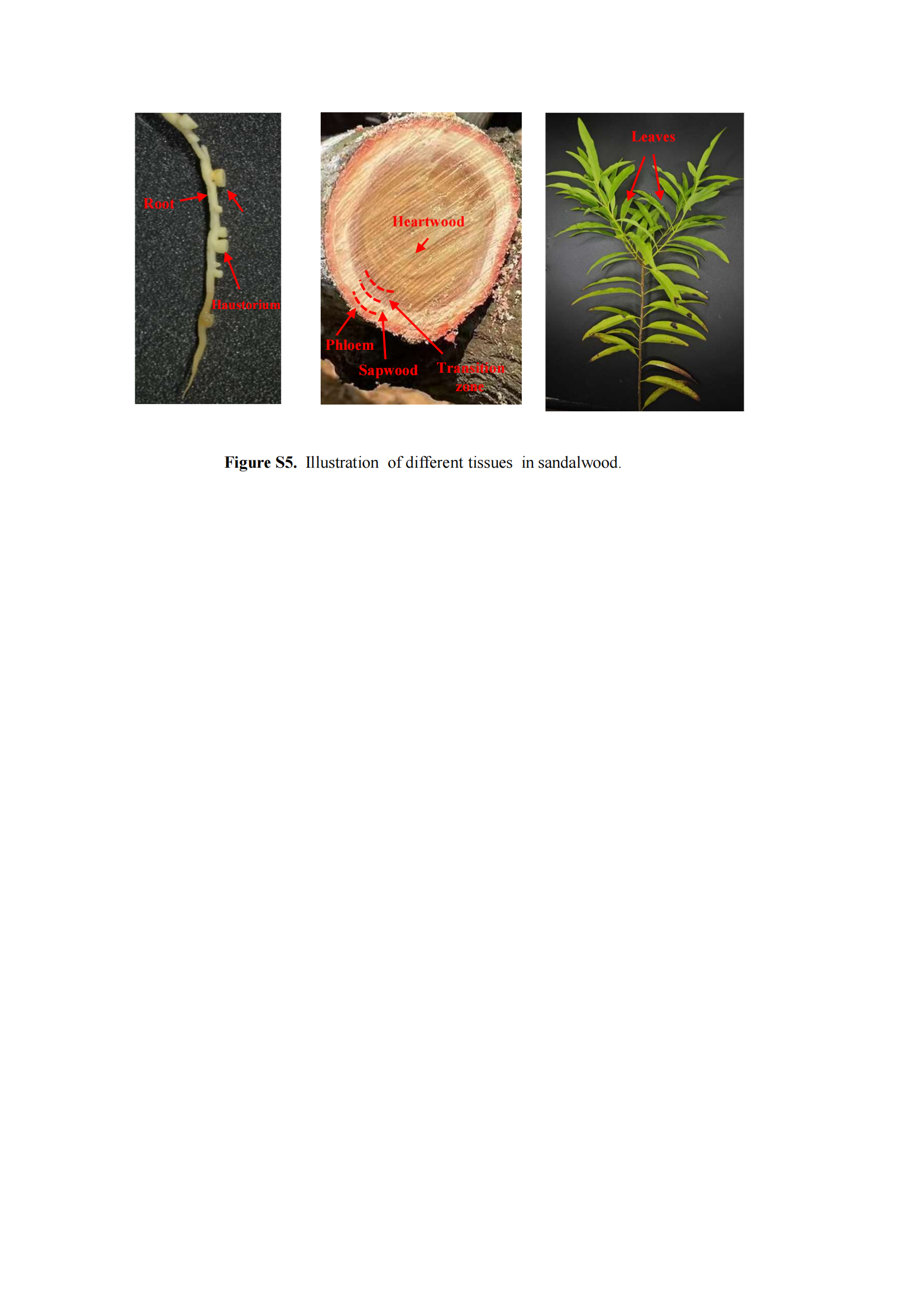

Supplement: Web_Material_uhae347 [file web_material_uhae347.zip › Fig S5.tif]
