# Supplementary material for: The AREB transcription factor SaAREB6 promotes drought stress-induced santalol biosynthesis in sandalwood
Source: Hortic Res. 2024 Dec 17;12(3):uhae347. doi: 10.1093/hr/uhae347 (PMC11890025; doi:10.1093/hr/uhae347)
Supplement: Web_Material_uhae347 [file web_material_uhae347.zip › Table S3.doc]

**Table S2.**  Primers used for qRT-PCR, cloning of *SaAREB6*, SaCYP736A167, yeast one-hybrid assay, protein expression, and identification of transgenic calli.

| Gene model | Gene Name | Sequences | |
| --- | --- | --- | --- |
| **Primers used for qRT-PCR** | | | |
| Sa3G15050 | *SaAREB6* | TCAACATTACAAGGTGGCGC | TGTATGGAACCGGTGACACA |
| Sa8G10740 | *SaIPPI* | TGCTTCCCACAAAATTCGCA | GTCTTCGAACATGAGACGCC |
| Sa6G08320 | *SaGDS* | TCAGTTATGGGACGTTGCCT | TCCTGGGCTACTATCCGTCT |
| Sa8G22960 | *SaFDS* | CACTCGGTTGGTGCATTGAA | ATCAGACCAACCTCAGGCAA |
| Sa3G10690 | *SaSSY* | TTCATGCGACATCTCTCCGA | CAGCAGCCCTTTTACGTTGT |
| Sa1G21730 | *SaCYP736A167* | TTATGGTGATCGGGCGAAGT | TATGCGCCGAGTTAATCCCT |
| Sa9G03100 | *Actin2/7* | TGCTCTTCCCCATGCCAT | AACAATTTCCCGCTCAGCAG |
| **Primers used for cloning of *SaAREB6*** | | | |
| Sa3G15050 | *SaAREB6* | ATGAATTTGAGGAATTTCGGGA | CCATGGCCCCGTCTGTGTCCG |
| **Primers used for Y1H** | | | |
| Sa3G15050 | *SaAREB6* | GCCAGTGAATTCCACATGAATTTGAGGAATTTCGGGA | CGTATCGATGCCCACTTACCATGGCCCCGTCTGTGTCCG |
| Sa1G21730 | *SaCYP736A167* | AATTCCCGGGGAGCTCGAGCTCGTTGCATTTTAACAGG | GCGAACGCGTGCGGATCCATACTTGACTTAGGAG |
| **Primers used for** **identification of transgenic lines** | | | |
|  | *SaAREB6#*OE | ccgtaaagactggcgaaca | ctcttcggcactccaaatgt |
|  | *SaAREB6#*RNAi | acaatcccactatccttcgca | ccatcaatccagacctccca |
|  | *SaCYP736A167#RNAi* | aatggtgaatttgagcgcga | cccgggctctgtaactatca |
| **Primers used for proteins expression in EMSA and MST** | | | |
| Sa3G15050 | *SaAREB6* | GACTTTGAGGCAACTCTAGAATGAATTTGAGGAATTTCGGGA | CTTCACGATACACCCGGGCCATGGCCCCGTCTGTGT |
| **Probe used for EMSA** | | | |
| Sa1G21730 | SaCYP736A167 | R1: ATTCTCGAACACGTGTTTGAC | |
|  |  | R2: GAAGCACGTGAATTTGGCA | |
|  |  | MutR1: ATTCTCGAACCCGGGTTTGAC | |
|  |  | MutR2: GAAGCCCGGGAATTTGGCA | |
|  | **Sequence for RNAi transgenic plants** | | |
| Sa3G15050 | *SaAREB6* | aagacctaattctatcaatgttttaggccttgggtttcaacagattgatgggaggtctggattgatgggcaatcgaatattggagagtaataatcattttgctatgcaatctcttaatttaccattgaatgtcaatggggtaacaccttcccagcagcagcagcaacagcaattgttttcccagcaaaatcccacttacacatctcccatgtctttaccaactagtgctcaactgggcagtcctggaatt | |
| Sa1G21730 | SaCYP736A167 | cgaaatggtgaatttgagcgcgagggtggcgaacgtcattgaaaacatggtgtgccttatggtgatcgggcgaagtagcgatgagaggtttaagctaaaggaggtcatccaggaggcagcgcagttggcgggagctttcaatataggggattatgttccattccttatgccccttgacctacagggattaactcggcgcataaagtcaggaagtaaagctttcgacgacatcttggaagtcataatcgacgagcacgtgcaagacattaaggaccatgatgatgaacaacatggagacttcattgatgt | |
